# Acceptability of, and preferences for, remote consulting during COVID-19 among older patients with two common long-term musculoskeletal conditions: findings from three qualitative studies and recommendations for practice

**DOI:** 10.1186/s12891-022-05273-1

**Published:** 2022-04-02

**Authors:** Zoe Paskins, Laurna Bullock, Fay Manning, Simon Bishop, Paul Campbell, Elizabeth Cottrell, G. P. Partner, Clare Jinks, Melanie Narayanasamy, Ian C. Scott, Opinder Sahota, Sarah Ryan

**Affiliations:** 1 School of Medicine, Keele University, ST55BG, Keele, UK; 2 Haywood Academic Rheumatology Centre, Haywood Hospital, Midlands Partnership NHS Foundation Trust, ST67AG, Stoke-on-Trent, UK; 3 University of Exeter Medical School, EX12LU, Exeter, UK; 4 Centre for Health Innovation, Leadership and Learning, University of Nottingham, Nottingham, NG8 1BB UK; 5 Wolstanton Medical Centre, Newcastle-under-Lyme, ST58BN UK; 6 Organisational Behaviour and Human Resource Management, Nottingham University Business School, NG81BB, Nottingham, UK; 7 Department of Health Care of Older People, Nottingham University Hospitals NHS Trust, NG72UH, Nottingham, UK; 8 School of Nursing, Keele University, ST55BG, Keele, UK

**Keywords:** Telemedicine, Remote consultation, Osteoporosis, Rheumatoid arthritis

## Abstract

**Background:**

Guidance for choosing face-to-face vs remote consultations (RCs) encourages clinicians to consider patient preferences, however, little is known about acceptability of, and preferences for RCs, particularly amongst patients with musculoskeletal conditions. This study aimed to explore the acceptability of, and preferences for, RC among patients with osteoporosis and rheumatoid arthritis.

**Methods:**

Three UK qualitative studies, exploring patient experiences of accessing and receiving healthcare, undertaken during the pandemic, with people with osteoporosis and rheumatoid arthritis. Study team members agreed a consistent approach to conduct rapid deductive analysis using the Theoretical Framework of Acceptability (TFA) on transcripts from each data set relating to RC, facilitated by group meetings to discuss interpretations. Findings from the three studies were pooled.

**Results:**

Findings from 1 focus group and 64 interviews with 35 people were included in the analysis. Participants’ attitudes to RC, views on fairness (ethicality) and sense-making (intervention coherence) varied according to their needs within the consultation and views of the pandemic. Some participants valued the reduced burden associated with RC, while others highly valued non-verbal communication and physical examination associated with face-to-face consults (opportunity costs). Some participants described low confidence (self-efficacy) in being able to communicate in RCs and others perceived RCs as ineffective, in part due to suboptimal communication.

**Conclusions:**

Acceptability of, and preferences for RC appear to be influenced by societal, healthcare provider and personal factors and in this study, were not condition-dependant. Remote care by default has the potential to exacerbate health inequalities and needs nuanced implementation.

**Supplementary Information:**

The online version contains supplementary material available at 10.1186/s12891-022-05273-1.

## Background

The COVID-19 pandemic led to the widespread adoption of remote consultations [1], (telephone, video, or online) [2]. Although the threat of COVID has changed over the course of the pandemic, the traditional healthcare model of in-person face-to-face appointments is increasingly viewed as unsustainable. Whilst remote consultations offer many potential advantages to patients and healthcare services, they are unlikely to be suitable for all [3]. Older adults living with frailty or those managing daily challenges with sight, cognitive, hearing or mobility impairment, may face additional barriers to engage with remote consultations [4]. A move to remote consulting by default [5] therefore has potential to increase digital exclusion for older adults [6]. With this in mind, clinicians identified concerns around which patients to select for remote consultations, in the context of clinical risk [2].

Various organisations have produced guidance on remote consulting, including the British Society of Rheumatology which highlight the importance of seeking patient preferences for consulting modality [7–9]. The little available evidence about patient acceptability of remote consultations, suggest preferences vary, relative to personal and contextual factors, such as the type of technology, patient ability and capability and the type of illness (including presence or absence of symptoms) [10]. Furthermore, it’s likely that patient views have changed over the course of the pandemic: from positive depictions of efficiency and reduced infection risk to negative narratives related to safety (e.g. missing diagnoses), inequality, and lack of choice [11].

Existing syntheses of evidence about the evaluation of remote consultations among people with long term conditions do not include patients with musculoskeletal disorders [12, 13]. Two recent reports suggest, among patients with musculoskeletal disorders, satisfaction with remote consultations is generally high, however, an overriding preference for face-to-face consultations persists [14, 15], raising questions that necessitate further exploration using qualitative methods [10].

The aim of this paper is to report the acceptability of, and preferences for, remote consultations among patients with two common long-term musculoskeletal conditions.

## Methods

Findings were pooled from three qualitative studies undertaken within our Academic Rheumatology group during 2020–2021. Each study was exploring patient experiences of accessing and receiving healthcare for their physical health. However, the studies varied with respect to their populations (people with (rheumatoid arthritis (RA) and osteoporosis) [16] and their healthcare encounters (which were variably pre- or intra-COVID-19 pandemic, patient- or professional-initiated and within both primary and secondary care settings) allowing comparisons on acceptability and preferences between symptomatic and non-symptomatic conditions, consideration of issues within a patient journey (i.e. point of diagnosis vs follow-up) as well as an examination of how views have changed over the pandemic.

Each study had ethical approval: iFraP (improving fracture prevention study [16]; North West - Greater Manchester West Research Ethics Committee, REF: 19/NW/0559), Blast Off (BO; Bisphosphonate aLternAtive regimenS for the prevenTion of Osteoporotic Fragility Fractures; North West - Preston Research Ethics Committee, REF: 19/NW/0714), and ERA (Exploring people with RAs’ experience of the pandemic; Camden and Kings Cross Research Ethics Committee, REF: 20/HRA/3406). The aims, data collection methods, sampling and recruitment and analysis for each study are detailed in Table 1 and Additional file 1.

**Table 1:** Description of contributing studies

Study name, funder(s)	Study authors	Aim	Design	Context and eligible population	Number of participants^a^	Dates of data collection
**Improving Fracture Prevention Study (iFraP),** NIHR, ROS, Haywood Foundation	ZP EC LB FM CJ	To explore experiences of FLS^b^ appointment and subsequent GP consultations and, their preferences for using decision aids in face-to-face or remote consultations	Focus group and semi-structured interviews (latter was an ethically approved change to protocol due to pandemic)	Men and women aged 50+ who had recently received a new diagnosis of osteoporosis and recommendations for treatment in the West Midlands region of UK	5/8	March –May 2020
**Blast Off Study** (Bisphosphonate aLternAtive regimenS for the prevenTion of Osteoporotic Fragility Fractures)**,** NIHR	ZP EC FM SB MN OS	To explore participants’ experience of receiving bisphosphonate treatment for osteoporosis, including their consultations with healthcare professionals.	Telephone semi-structured interviews	Men and women aged 18+ in the UK who had taken or received a bisphosphonate within the last 24 months (at the time of invitation to participate) for the prevention of fragility fractures	15/70	June–August 2020
**Experiences of patients with RA, during the coronavirus pandemic** (ERA), Haywood Foundation	SR PC FM ZP	To explore the experience of living with RA on physical, psychological and social wellbeing during the pandemic, including their experience of receiving healthcare	Three longitudinal semi-structured interviews	Men and women with RA in West Midlands region of UK	15/15	Interview 1 16th Sept-23rd Nov 2020 Interview 2 11th Jan - 27th Jan 2021 Interview 3 27th Apr - 29th June 2021

*ZP* Zoe Paskins, *EC* Elizabeth Cottrell, *LB* Laurna Bullock, *FM* Fay Manning, *CJ* Clare Jinks, *SB* Simon Bishop, *MN* Malenie Narayanasamy, *OS* Opinder Sahota, *SR* Sarah Ryan, *PC* Paul Campbell, *GP* general practitioner, *FLS* Fracture Liaison Service, *UK* United Kingdom, *RA* Rheumatoid Arthritis, *NIHR* National Institute for Health Research, *ROS* Royal Osteoporosis Society

^a^Number of participants that contributed data to this study/total number of patient participants in contributory study

^b^ Fracture Liaison Services (FLSs) are typically nurse-led services which enact secondary fracture prevention in people aged 50 and over with low trauma fractures. The services involve assessment of bone health, assess for osteoporosis and make recommendations for osteoporosis treatment. Participants had attended an FLS within secondary care

### Analysis

This study used the Theoretical Framework of Acceptability (TFA) [17], developed to inform the understanding of acceptability of complex interventions (defined for our context as a remote consultation), and consists of seven constructs (Table 2), and previously used to explore the acceptability of consultations [18].

**Table 2:** Theoretical Framework of Acceptability

TFA Domain	Description
Affective Attitude	the emotions elicited by an intervention
Intervention Coherence	the extent to which an intervention makes sense
Perceived Effectiveness	the perceived extent to which intervention will achieve purpose
Burden	the amount of effort required to participate in an intervention
Self-Efficacy	an individual’s confidence that they can perform the behaviour(s) required to participate in the intervention
Opportunity Costs	the extent to which benefits, profits, or values must be given up to engage in an intervention
Ethicality	the extent to which an intervention has a good fit with an individual’s values

*TFA* Theoretical Framework of Acceptability

**Fig. 1:**
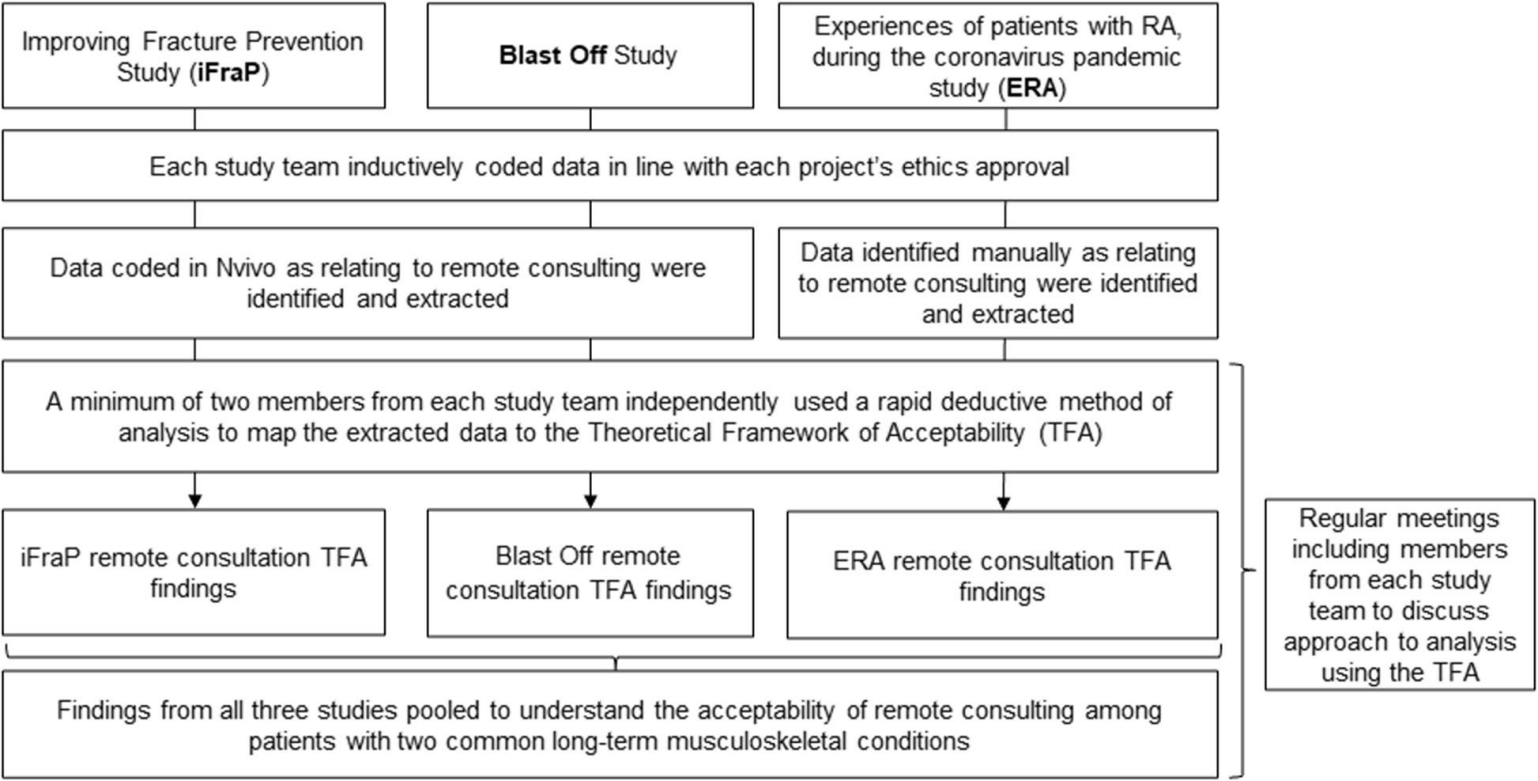
Overview of methods and analysis

Each individual team conducted separate analysis in line with each project’s ethics approval. Each study team conducted inductive coding and identified descriptive codes relating to remote consultation experiences. Thereafter, data identified as about remote consulting in the previous inductive coding was mapped by each team to the TFA using a rapid deductive method of analysis. Two studies (iFraP and BO) used NVivo to identify original data coded as relating to remote consultation experience, under one of the seven TFA domains. The third study did the same process manually. During this process, members from all study teams had regular meetings to discuss their approach to coding to ensure consistency of approach. Within each study team, a minimum of two authors completed the coding, working independently. Finally, the findings of all three studies individual and independent analysis was pooled (Additional file 1). An overview of this process is provided in Fig. 1.

## Results

Findings from 1 focus group and 64 interviews with 35 people are included in the final analysis (iFraP – 5; BO – 15; ERA – 15). Participant characteristics are detailed in Table 3.

**Table 3:** Participant characteristics

Study name, funder(s)	Number of males: females	Ethnicity	Employment	Age	Disease duration / treatment initiation	Participant experience of remote consultations
**Improving Fracture Prevention Study (iFraP),** NIHR, ROS, Haywood Foundation	1:5	White	1 working 1 retired 3 unknown	Mean 68 (range 60–71)	Diagnosed at their recent FLS appointment	Participants had received telephone consultations either pre or early pandemic in both FLS and primary care about osteoporosis
**Blast Off Study** (Bisphosphonate aLternAtive regimenS for the prevenTion of Osteoporotic Fragility Fractures), NIHR	0:15	Data not collected	4 working (2 employed; 2 doing voluntary work) 5 retired 6 unknown	Mean 67 (range 58–75)	Initiated bisphosphonate treatment (oral or IV) between 1997 and 2020	Telephone consultations with primary care clinicians and specialists about osteoporosis
**Experiences of patients with RA, during the coronavirus pandemic** (ERA), Haywood Foundation, NIHR	6:9	White	4 working 1 not working 10 retired	Mean 64 (range 46–78)	Disease duration mean 22.2 years (range 1.5–46 years)	Telephone consultations with specialists in secondary care and primary care about RA

*FLS* Fracture Liaison Service, *NIHR* National Institute for Health Research, *IV* intravenously, *ROS* Royal Osteoporosis Society, *RA* Rheumatoid Arthritis

Findings are discussed below relating to each domain of the TFA, along with a discussion of the key factors that were associated with variability in acceptability (such as condition and timing of the pandemic).

### Intervention coherence

Intervention coherence concerns the extent to which remote consulting made sense to participants. Sense making was influenced by participants’ expectations and needs for the consultation. Some participants, including participants with osteoporosis and participants with RA, felt that remote consultations made sense and were appropriate if they had need for: simple information, routine follow up and monitoring, knew their clinician and felt well, or wanted a quick response to a question or uncertainty.

*because I was ok, a telephone consultation was fine* (ERAP14–1).

In other circumstances, such as, receiving a new diagnosis for participants with osteoporosis, being unwell, requiring examinations or interventions for participants with RA, or if participants wanted to discuss a concern, e.g. a medication in depth, remote consultations were not deemed to make sense.

*I’ve had flare ups my hands have swollen up so much that the top of your hands they can double the size … I had just a telephone and I was like that I’d be saying ‘look I need some help, you need to see my hands I need more than just a telephone consultation* (ERAP14–1).

*to be given a diagnosis over the phone was atrocious. And I know now that lots of people have that experience* (B017p).

For others, remote consultations only made sense in the context of the COVID-19 pandemic due to infection risk.

*cause of the circumstances I think that it’s the safest way to do it and the only way to do it, and we haven’t got really much choice* (iFraP3).

Although participants across all studies had long term conditions, remote consultations appeared to make sense most to those who felt well and were able to ‘make use’ of this technology. It made less sense to people who felt building a new caring relationship was important, had significant concerns, or who had significant physical symptoms.

### Perceived effectiveness

Participants considered the extent to which remote consultations could be effective in reaching their goals. Remote consultations were deemed as being effective to enable timely solution to a problem.

*I mean for me it was more convenient because I could have the conversation I needed to have quickly and efficiently* (ERAP1–1).

However, some participants noted that effectiveness of the information exchange may be limited because of information not being discussed in the same amount of depth as it would be face-to-face, or that discussions may not be as frank or honest.

*I think there is a danger that you maybe under playthings on the phone, it’s quite difficult to really tell people how you are feeling* (ERAP1–1).

*you don’t think of the right range of questions when you’re on the phone* (B073p).

External factors could also impede the quality of communication.

*I probably wasn’t hearing her properly either because we were outdoors and the wind interferes with a phone conversation. It was the wrong place to be having a conversation like that* (B017p).

Use of the telephone was mainly seen as effective when the goal was to exchange relatively emotionally neutral, explicit information, whereas it was seen as less appropriate when communicating more emotionally loaded and open-ended information that required shared tacit understanding. The telephone was also described as less useful for receiving detailed explanations about challenging concepts which might require the use of pictures, written information and resources.

The perceived effectiveness of remote consultations also appeared to be affected by the perceived strength of relationship with the healthcare provider.

*I think if it was the first appointment or an early appointment that would be very difficult on both parts you know for the consultant as well really* (ERAP1–2).

Remote consultations were not perceived as effective in building therapeutic relationships.

*I don’t know what that person at the other end of the phone is doing are they frowning, is it a bit of a sneer … Relationships come from interpersonal interaction and that happens in a similar space, it doesn’t happen at a distance* (ERAP09–3).

For those with physical symptoms, telephone consultations were sometimes felt to be ineffective.

*you can’t replace the face-to-face where they have to look at your joints and feel your joints and see what movements are hurting* (ERAP1–3).

Although perceptions of how effective remote consultations were related to nature of the patient’s problem and or diagnosis, and the need for physical examination, perception of effectiveness were also influenced by other external factors such as the relationship with the healthcare provider, the timeliness of the consultation (enabling quick problem resolution) and inter-personal factors relating to communication.

### Self-efficacy

Self-efficacy concerns the extent to which participants felt confident in engaging with remote consultations. Some participants described how their skills and confidence in using technology had increased over the course of the pandemic.

*After the last few weeks of Zoom and WhatsApp video I don’t think I’d mind. Prior to that I might have said that I preferred the telephone, but I don’t think I’d mind now* (iFraP4)

However, others described their difficulties in being able to communicate effectively over the phone. This could be due to factors associated with the patients’ health conditions, but was also mediated by external factors, such as interference on mobile phone, emotions, such as feeling anxious or being underprepared or other barriers to communication. This confidence in communicating appeared to influence their overall preferences for remote consultations.

*it’s hard to explain to somebody how things are aching and it hurts when you move* (ERAP8–1)

*I’m of sound mind as I can explain, but I’m sure there’s loads of people out there that can’t explain exactly what their situation is and it has to be a visual thing* (ERAP6–3)

*I’m getting there with phones. If it actually rings I go into a panic.* (iFraP3)

Reduced confidence in communicating over the phone was described by participants across all three studies, and by participants of a range of ages and employment status.

### Burden

Burden concerns the ‘work’ participants encountered in relation to remote consultations. Participants who were comfortable with remote consultations reflected on the benefits associated with less travel, shorter waiting times, and no need for time off work, resulting in perceived efficiency for some participants.

*you haven’t got well the time off work, you haven’t got the travelling up there, the waiting around with people and all that you know it’s a matter of a telephone call that you can do in your break you know I just come off the computer and speak to her* (ERAP6–1).

Patients also valued the benefits associated with not having to undergo sanitisation procedures or use personal protective equipment.

*The situation of having to wear a mask and wear gloves and sanitise and all this that and the other, that’s gone so I think it puts people able to take a sort of deep breath and a sigh to think ‘well I haven’t got to go through all this rigmarole’* (ERAP2–1).

Although most participants described lower burden, some did describe remote consultations as inconvenient due to the wide time window associated with telephone calls.

*I wasn’t hugely thrilled that he rang me on my mobile and I was on a day out with a friend … and walking round ... suddenly get this information that perhaps was a bit suboptimal!* (B012p).

### Opportunity costs

Opportunity costs concern the extent to which other values or benefits have to be given up in order to participate in remote consultations. For example, remote consultations meant giving up the opportunity to receive a physical examination. For participants with RA, this was described as important not just to see the joints, but because of touch and to demonstrate the severity of the patient’s condition.

*You need to be seen by a consultant cos if they’re actually touching your feet or looking at your hands they actually need you in person* (ERAP3–3).

*they can see you and how bad you are* (ERAP8–1).

Participants with osteoporosis also valued the in-person nature of communication. Remote consulting meant, in the perception of the patient, if attending with a new problem or if they had uncertainty, giving up the opportunity for the clinician to see the patient’s (and their family member/caregiver’s) body language and expressions meaning the doctor could ‘read’ what was wrong.

*Face-to-face can be quite important can’t it because you know the doctor, you walk into the surgery and the doctor immediately can read from the way in which you’re conducting yourself* (iFraP2).

*you can tell an awful lot from a person’s face* (B033p).

In person presence was also associated with a feeling of being cared for.

*maybe it’s part of the human psyche but if you’re seeing a person face-to-face you do feel like you’re being more looked after … You do feel like you’re more of an individual and getting more of a service just due to the fact that you’re there* (ERAP4–3).

Participants traded reduced burden vs opportunity costs depending on their personal situation (e.g. working, or other responsibilities) and the nature of their healthcare problem for which they were consulting.

### Ethicality

Ethicality concerns the extent to which remote consultations are viewed as ‘fair’. Even during the pandemic, access to face-to-face consultations was viewed as a fundamental ‘right’.

*I rang the GP back and again they gave me another telephone call. At no point did I see the GP and all they reiterated was ‘yes, it’s osteoporosis, Alendronic Acid is what you need and there is a prescription ready and waiting for you’ … So I didn’t take it up at that point. I started to get quite upset about it because I felt that I’d been diagnosed without a lot of support.* (B032p).

Some participants across two studies, considered the wider population, commenting that although their experience had been good, remote consultations may not be suitable for all leading to unequal access to care.

*My mother wouldn’t cope (…*) *we’re all being expected to be computer literate and many people over 80 aren’t and are feeling slightly excluded* (iFraP4).

Conversely, some considered the move to remote consultations to be a fairer allocation of care, with the perception that quicker telephone consultations, even if less effective, meant more people could have access to consultations more quickly.

*if it could be dealt with over the phone and a lot of things can or video … it’s not as good but if it’s going to be quicker rather than waiting 6 months to see somebody … if you can have a quick video consultation it’s much better for everybody* (ERAP1–3).

### Affective attitude

Affective attitudes concerns the emotions and feelings described in relation to remote consulting. Positive emotions, associated with satisfaction with the process, and feelings of safety, were elicited particularly during the pandemic.

*I’m quite happy doing this on the phone. I’d probably be equally happy doing it by video.* (iFraP4).

*I feel safer at home, so I would prefer to have a phone call* (ERAP15–1).

More negative emotions were related to feelings of frustration and feeling isolated.

*you can feel cut off* (ERAP9–1).

*I did not like it … as soon as the opportunities for face-to-face contact re-arrive, I believe we should firmly go back to that. I have always been hostile to the telephone call* (ERAP9–2).

In some instances, a phone call itself elicited feelings of anxiety, possibly related to hurriedness.

*If I’d been sat in the GP’s surgery with him, I think I would have been a bit calmer and more focused, than talking to me on the phone like that.* (B073p).

Both positive and negative emotions were elicited across all studies and were related to perceptions of perceived effectiveness but were also related to other personal factors related to sense making (intervention coherence) and self-efficacy.

## Discussion

**Fig. 2:**
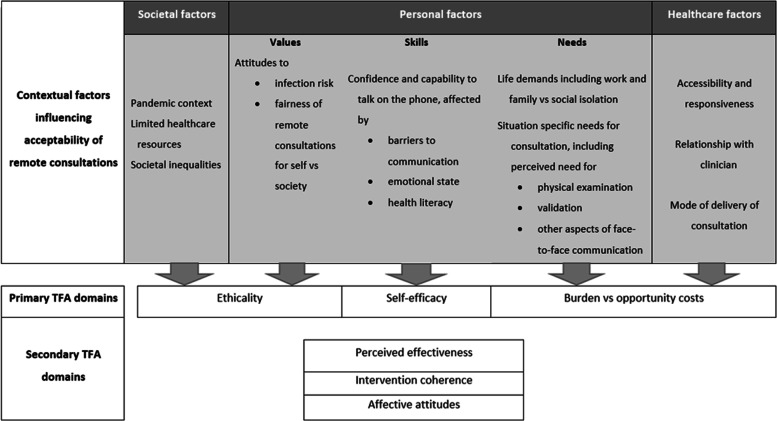
Summary of findings relating to TFA domains

In patients with osteoporosis and RA, acceptability of, and preferences for, remote consultations appear to be influenced by a wide range of societal, healthcare provider and personal factors (Fig. 2). In people who value the reduced burden associated with remote consultations and have high self-efficacy, preferences for remote consultations seem to depend on their characteristics of their situation, relating to their expectations and needs for the consultation, and the extent to which their healthcare provider could meet those needs. However, in people who do not value the reduced burden, had low self-efficacy, or did not perceive remote consultations as effective or fair, acceptability, which was low, appeared to be more of a fixed belief, with preferences expressed to avoid remote consulting at all costs. Although the reasons underlying acceptability in some instances were not condition-dependant, some participants with RA who were symptomatic and perceived a need for physical examination expressed a preference for face-to-face consultations. However, so too did some participants with osteoporosis who valued in-person and non-verbal communication. Acceptability may have been in part context-dependant, with participants perceiving remote consultations as making more sense and being ‘fairer’ earlier in the pandemic. Some participants with both conditions perceived that communication in remote consultations could be suboptimal, and participants with RA described a feeling of being more ‘cared for’ in face-to-face encounters.

### Comparison with existing literature

Recent studies reporting the preferences and experience of remote consultations for patients attending therapies in orthopaedics and appointments in outpatient rheumatology, identified similar findings to ours, relating to variable preferences depending on patient expectations, current situation, and capacity [19, 20]. Interestingly, one paper also reported the importance of physical touch in determining preferences for face-to-face contact [20]. Since we conducted our analysis, a mixed methods study examining patient and clinician view of telemedicine in rheumatology has been published [21]. This much larger study which included patients with rheumatoid arthritis and lupus identified telemedicine was perceived to be associated with increased misdiagnoses, inequalities and barriers to accessing care. Similar to our study findings, concerns about the impact of telemedicine on building trusting relationships were raised, and the importance of physical examination [21]. However the inclusion of patients with osteoporosis in our study means that we have been able to explore the views of people with potentially asymptomatic conditions; furthermore our use of the TFA has led us to examine further the impact of telemedicine on people’s lives, moving away from a purely medical focus.

Importantly, our findings demonstrate the difficulty that some patients have in communicating on the telephone. Recently, the Patient Information Forum reported that in the UK, 1.7 million people are unable to explain symptoms or feelings on the phone [22]. This is likely to impair the quality of patient-clinician shared decision-making and widen existing health inequalities and these challenges are likely amplified by low health or digital literacy, hearing, visual, or cognitive impairment or language barriers [23–26], commonly experienced by older adults [6]. Studies have continued to highlight patient and clinician preferences for face-to-face consulting, with evidence that clinicians want to see and examine patients with RA [21, 27–29].

In our study, even in participants who did not report low confidence in talking on the phone, perceptions were identified that remote consultation communication was less effective and more hurried. A previous quasi-experimental study identified that some elements of consultation quality were lower in video or telephone consultations, and that although video consultations showed more evidence of rapport building, both video and telephone consultations were less ‘information rich’ than face-to-face encounters [30]. Furthermore, remote consultations were less likely to feature discussion of the problem in the psychosocial context, which has important relevance for our findings given the reports of isolation and anxiety associated with remote consultations.

In describing the TFA, Sekhon et al. indicated that the extent to which the domains of acceptability cluster or are interrelated is an empirical question [17]. In this study, we identified considerable overlap between domains. Perceived effectiveness was conceptualised a ‘secondary domain’ meaning that patients’ perceived effectiveness of remote consulting was dependant on their perceptions of self-efficacy, ethicality, burden and opportunity costs. In previous qualitative research using the TFA, we also identified affective attitudes and intervention coherence as ‘secondary domains’ [18, 31]. The TFA has been criticised for excluding social and environmental factors [32, 33], although we were able to incorporate wider macro and meso level influences into our model (Fig. 2) demonstrating how these issues interrelate with the TFA domains.

### Strengths and limitations

We pooled findings from three qualitative studies, which enabled us to explore the sources of variability in acceptability of remote consultations across a larger sample. Pooling qualitative studies to ‘scale up’ facilitates generation of cross-contextual understandings and explanations [34]. In order to conduct and quickly disseminate findings which immediate implications for practice, we conducted a rapid theoretically-informed deductive analysis within each study. More in-depth findings may have been identified from a more inductive approach. However, evidence suggests rapid analysis does not result in inferior findings and use of a large team may increase the robustness of analysis [35, 36].

Our study populations included a predominance of older females, and people with experience of telephone, rather than video consultations. Two of the three contributory studies included specific questions about remote consulting in their topic guides, meaning that in the BO study, contributory findings were only from participants who volunteered information about remote consultations. The majority of findings were from the ERA study where participants were describing contacts with both primary and secondary care, however, we did not find acceptability varied according to healthcare setting.

### Implications for policy, practice and research

**Fig. 3:**
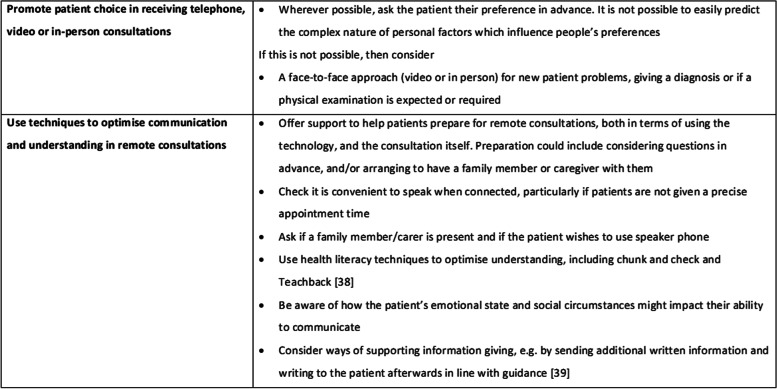
Recommendations for clinical practice

At a policy level, these findings add to evidence to suggest that ‘remote care by default’ is highly likely to exacerbate health inequalities and therefore needs nuanced implementation [5]. Our findings need to be balanced with other evidence about the safety and clinical effectiveness of remote consultations and considered in conjunction with recommendations for practice from the clinician perspective that include guidance on clinical or service factors which might influence triage to remote or face-to-face consultations. However, exploring the patient perspective has led to three recommendations for practice. First, due to the complex and interactional nature of personal and contextual factors which influence acceptability of remote consultations, patient preferences cannot be easily predicted, and therefore we suggest the most effective way of identifying preferences for remote consultations would be to ask patients (or, if appropriate, their family member/carer) prior to specific encounters [37]. Second, for a range of reasons, communication is more likely to be sub-optimal in remote consultations and specific strategies should be employed to mitigate this risk (Fig. 3) [38, 39]. Further research is needed to explore the role of video consultations in this context and how the quality of clinician-patient communication can be optimised in remote consultations. Third, the perception of the importance of physical examination for some populations, particularly those with RA is highlighted. The extent to which further support with remote consultations, such as patient reported outcome measures collected prior to appointments, can overcome perceived shortfalls of telephone consults remains to be seen.

## Conclusions

In older adults with osteoporosis and RA, acceptability of, and preferences for remote consultations are influenced by a wide range of factors which are not easy to predict and cannot be distilled to personal characteristics, contexts, or conditions. For that reason, offering patients choice of consulting modality, rather than offering remote care by default, appears to be the most patient-centred approach in deciding whether remote or face-to-face consultations are most appropriate, if this is possible in the clinical and service context. Our findings suggest communication and information exchange may be less effective in remote consultations and further research is needed to explore this further.

## Supplementary Information

**Additional file 1: Table S1.** Additional information about contributing study methods. **Table S2.** Pooled study findings.

## Data Availability

All relevant data generated or analysed during this study are included in this published article supplementary information files. Complete transcripts are not available to protect participant anonymity.

## Abbreviations

FLS: Fracture Liaison Service
NIHR: National Institute for Health Research
ROS: Royal Osteoporosis Society
RA: Rheumatoid Arthritis
TFA: Theoretical framework of acceptability
iFraP: Improving fracture prevention study
BO: Blast Off Bisphosphonate aLternAtive regimenS for the prevenTion of Osteoporotic Fragility Fractures
ERA: Exploring people with RAs’ experience of the pandemic
UK: United Kingdom
