# Supplementary material for: Acceptability of, and preferences for, remote consulting during COVID-19 among older patients with two common long-term musculoskeletal conditions: findings from three qualitative studies and recommendations for practice
Source: BMC Musculoskelet Disord. 2022 Apr 2;23:312. doi: 10.1186/s12891-022-05273-1 (PMC8976169; doi:10.1186/s12891-022-05273-1)
Supplement: Supplementary file 1 — Additional file 1: Table S1. Additional information about contributing study methods. Table S2. Pooled study findings. [file 12891_2022_5273_MOESM1_ESM.docx]

**Additional file 1**

**Table S1: Additional information about contributing study methods**

Of the three studies, one had previously published a protocol^1^ and was registered with Research Registry (REF: researchregistry5041), and another had published a prior scoping literature review^2^ but not a protocol.

1. Paskins Z, Bullock L, Crawford-Manning F, et al Improving uptake of Fracture Prevention drug Treatments: a protocol for development of a consultation intervention (iFraP-D). BMJ open. 2021 Aug 1;11(8):e048811. http://dx.doi.org/10.1136/bmjopen-2021-048811

2. Paskins Z, Crawford-Manning F, Cottrell E, et al Acceptability of bisphosphonates among patients, clinicians and managers: a systematic review and framework synthesis BMJ Open 2020;10:e040634. h[ttp://dx.doi.org/10.1136/bmjopen-2020-040634](http://dx.doi.org/10.1136/bmjopen-2020-040634)

| **Study name** | **Author role and reflexivity** | **Sampling and Recruitment** | **Aim and Data Collection procedures** | **Analysis** | **Patient and public involvement** |
| --- | --- | --- | --- | --- | --- |
| **Improving Fracture Prevention Study** (iFraP) | LB interviewed – experienced qualitative researcher, unknown to participants | Participants included patients that had recently attended an FLS consultation and received a treatment recommendation. Two FLSs in the UK West Midlands region recruited patient participants. The FLSs were provided with study information packs to hand to patients during or after their FLS appointment which included an invitation letter, participant information sheet, reply slip and return envelope. | Exploration of current practice in relation to communication about fracture prevention drug treatment, anticipated barriers and facilitators to the iFraP intervention and perceived training needs. Topic guides were co-developed with PAG members and informed by the TFA, TDF and NPT.  Two patient focus groups were planned. The first face-to-face focus group included n=4 with n=1 discussing their experience of remote consulting. Ethics approval was sought to replace the second focus group with patient telephone interviews (n=4) due to the COVID19 pandemic. | Interviews were digitally recorded, transcribed verbatim, and anonymised. A two-stage analysis incorporating the inductive coding of transcripts followed by a deductive exercise to map identified codes to the domains of the TFA, TDF and NPT. Analysis was conducted by LB with a sample of transcripts coded by ZP to explore the consistency of coding. Mapping to theoretical frameworks and interpretation of findings were discussed between experienced qualitative researchers (LB, ZP, CJ). | Two dedicated iFraP PAG meetings were convened. The first face-to-face meeting (n=2) co-designed topic guides to ensure that content was relevant and understandable. The second virtual PAG meeting (n=4) facilitated data analysis and provide interpretation of coding. |
| **Blast Off Study**  (Bisphosphonate aLternAtive regimenS for the prevenTion of Osteoporotic Fragility Fractures) | MN interviewed - experienced qualitative researcher, unknown to participants | Participants were recruited through an open advertisement in the Royal Osteoporosis Society’s Summer 2020 newsletter. Prospective participants contacted the research study team expressing their interest to participate, and were subsequently sent further study information (participant information sheet and reply form - the latter invited them to indicate when they had started bisphosphonate treatment so that the research team could confirm eligibility). | Semi-structured telephone interviews were conducted with participants exploring their perceptions and experiences of being diagnosed with osteoporosis, taking or receiving bisphosphonate treatments, and service factors. | Interviews were digitally recorded, transcribed verbatim, and anonymised. Interview data underwent intensive inductive open coding using NVivo, carried out by MN and SB. MN and SB noted any transcript which made reference to RC e.g. telephone diagnosis. Relevant data relating to RC from these transcripts was then mapped to the TFA constructs. | The interview schedule used with patients was developed in collaboration with the study’s steering group, which included individuals who were representative of patient and public engagement (PPE). Furthermore, the interview schedule was piloted with two PPE representatives. These approaches ensured that interview questions were relevant and understandable.  A virtual meeting was convened in December 2020 to enable SB and MN to present early work on the developing themes following inductive coding. Attendees included PPIE representatives, which enabled useful feedback and recommendations for focusing further analysis. |
| **Experiences of patients with rheumatoid arthritis, during the coronavirus pandemic** (ERA) | PC interviewed – unknown to participants | Participants with rheumatoid arthritis were recruited from a community hospital in Staffordshire. Participants were identified from a rheumatology clinical database. Participants were purposively sampled to ensure a representation of age, gender, diagnosis, shielding and non-shielding status. Potential participants were mailed an expression of interest letter inviting them to take part in the study. If a positive response was received, then a consent form and participant information sheet was posted to the participant. Patients who are unable or unwilling to provide consent were excluded from the study. | The longitudinal interviews explored changes to the impact on wellbeing and use of healthcare, at 3 points during the coronavirus pandemic. Separate topic guides were used for each interview and the content was influenced by events that occurred during the pandemic including perceptions of vulnerability and risk in interview 2 (relating to the use of being classified as clinically extremely vulnerable) and receiving vaccinations and behaviour change in interview 3. | Interviews were digitally recorded, transcribed verbatim, and anonymised. Interview data underwent intensive inductive coding by SR, noting any transcript which made reference to RC. | Two patient partners with RA, were involved in all stages of the research, including contributing to the study protocol, topic guide, public facing information (invitation letter, patient information sheet and consent form) and data analysis. The patient partners also took part in two pilot interviews and were able to provide feedback on improving the clarity of the questions in the topic guide. |
| FLS Fracture Liaison Service, TFA Theoretical Framework of Acceptability, TDF Theoretical Domains Framework, NPT Normalisation Process Theory, RC remote consultations, PAG patient advisory group, RA rheumatoid arthritis, PPIE patient and public involvement and engagement | | | | | |

**Table S2: Pooled study findings**

| **TFA domain and definition** | **Quotes** |
| --- | --- |
| **Intervention coherence**  *The extent to which the patient understands remote consultations and how they work* | *so because I was ok a telephone consultation was fine* (ERAP14-1)  *I have had a couple of consultations with the GP as well on the phone. But GP’s were moving more towards phone consultations anyway in my experience you know if they could* (ERAP1-1)  *I had a telephone call from a consultant which I found very useful, quite happy, didn’t need to see her face to face but then I feel there’s nothing wrong with me* (ERAP10-1)  *So some things you can talk about over the phone and sometimes it’s the reassurance you know it’s having somebody there that you can just say I’m struggling* (ERAP6-3)  *telephone consultations are fine, I mean I had to have a telephone consultation today purely because I want a referral with regards to my hip, so I think that’s absolutely fine however when I first went and wanted to know what was going on with my leg, I think they needed to see how I’m walking* (ERAP6-3)  *I find them fine. But I’m not ill, it might be a different thing if I ache all over and I desperately need to see someone* (ERAP10-1)  *I wouldn’t like it (a telephone consultation) to replace the face to face. I can see that for people who are stable and not having significant problems, I can see the value of keeping in touch via the telephone conversation however there have been times when I have gained a great deal from having the face to face* (ERAP9-1).  *so because I was ok a telephone consultation was fine* (ERAP14-1)  *So if it was just a standard like a run of the mill routine appointment I would be happy with that by phone, but if something had happened then I’d want them to actually physically see my joints, but I think it’s a bit of a mix and match really. Like discussing medication and stuff, that can all be done over the phone and results and things like that I’m absolutely fine with it that way* (ERAP3-2)  *there are discussions that can be had about medication that don’t need to be done in person* (ERAP3-1)  *well I didn’t mind it because of the situation in the summer as they weren’t seeing people at the [hospital] so it was a different situation, I wouldn’t like that all the time, the next one in a year’s time next summer when it’s due I would like to see someone but if we’re still in the middle of this pandemic obviously you wont* (ERAP10-1)  *I can understand why it’s done especially during the time of lockdown I would suggest it’s essential, so I’ve got to back away from the fact that I don’t like it to acknowledge that there is a need for it* (ERAP9-2)  *I don’t mind having a telephone appointment because under the circumstances I think the least we move around, cos I’m just as liable to take it up to [hospital] as I am to pick it up really, so I think the less we move around the better. If it was something that could be discussed over the telephone well that’s fine with me* (ERAP15-2)  *People that have a severe illness, severe condition who want an answer tomorrow, they want their answer tomorrow and I think the remote consultations* (ERAP2-1)  *but you know sometimes in the past when I’ve had flare ups my hands have swollen up so much that the top of your hands they can double the size and the thing is it can be like the biggest fattest sausage you’ve ever seen, I mean I used to look at them and think well who’s hands are these, and I’ve had to have a Steroid injection in my hand, now had I had just a telephone consultation and I was like that I’d be saying ‘look I need some help, you need to see my hands I need more that just a telephone consultation* (ERAP14-1)  *With my RA being stable you know someone phoning me and talking to me I’d say nice things and they could say ok keep in touch, if there’s a problem don’t hesitate to ring, I’d be happy with that however if the RA was up and down I would be thinking very differently.* (ERAP9-3)  *for reviews you know kind of 12 monthly reviews and the nurse or the GP looks at the info and decide whatever they’re looking out for is fine you know blood pressure, kidney function, cholesterol levels, if that is all fine then if you can do that from a remote standpoint and people are getting busier and busier these days too, so rather than going and losing an hours work and maybe have to ask the boss for time off etc. and go to the surgery, if that’s able to be done effectively via that kind of medium then yes there’s certainly value in that. It’s almost a hybrid situation that might be the way forward, a combination of the two where the routine stuff can be done remotely*. (ERAP4-3)  *You need to be seen by a consultant cos if they’re actually like touching your feet or looking at your hands they actually need you in person, but for all the other stuff all the kind of monitoring, that can be done by phone or email no problem* (ERAP3-3)  *I know treatment has changed the different way we have to do things i.e., having doctor’s appointments over the phone all the time, I don’t agree with some things just have to be seen (…) telephone consultations are fine, I mean I had to have a telephone consultation today purely because I want a referral with regards to my hip, so I think that’s absolutely fine however when I first went and wanted to know what was going on with my leg, I think they needed to see how I’m walking* (ERAP6-3)  *I would be willing to take part in either a telephone or a video appointment yes, the drawback I would think would be if they wanted to inspect your joints for inflammation which the doctors do they have a look, so they couldn’t really do that but they could see how you are going on with your present medication and if you got any problems or any advice that they can give to you and also that they’ve been in touch with you. So I would say that it was a plus point for them to get either in touch or a video but the drawback would be the fact that they couldn’t see if there was any inflammation or any problems with any of your joints* (ERAP7-3)  *It doesn’t offend me but I would resent it if that was the only means of doing it. With my RA being stable you know someone phoning me and talking to me I’d say nice things and they could say ok keep in touch, if there’s a problem don’t hesitate to ring, I’d be happy with that however if the RA was up and down I would be thinking very differently.* (ERAP9-3)  *for reviews you know kind of 12 monthly reviews and the nurse or the GP looks at the info and decide whatever they’re looking out for is fine you know blood pressure, kidney function, cholesterol levels, if that is all fine then if you can do that from a remote standpoint* (ERAP4-3)  *because I didn’t have any problems it was ok but I just wondered you know I suppose If I’d said to him ‘oh God my hands are in a right state’, he’d have said well come in and we’ll do something you know* (ERAP14-3)  *one of the lady doctors actually rang me at home to see how I was, which very good, good appointment but normally you would have your joints tested and they would see that everything was ok, but I suppose you do miss that and you think well I haven’t had that done now for well it’s two years over two years now, but on saying that I’m alright but I suppose if I was really poorly you’d be a bit concerned wouldn’t you only speaking on the phone.* (ERAP10-3)  *I mean the appointment that I had with the doctor [GP] on the telephone was my suggestion anyway because I thought it would be… I thought it was unnecessary to, you know, I just had a few questions which he could answer over the telephone, which he did and that was fine and I’d have done that anyway, even if the coronavirus issue hadn’t arisen I think (…) but on the other hand that’s really just when you want information isn’t it or guidance, if you’re ill which wouldn’t be the case with the fracture clinic (…) with this kind of thing [fracture liaison service consultations] where it’s a case of just asking questions and having a discussion, particularly when we now do have access to video links, increasingly, which are pretty good, then I would have thought it has great advantages* (iFraP2)  *I think both, but [follow up] could probably be done over the telephone* (iFraP1)  *I think face to face is still nicer but as an alternative, certainly for follow ups I think would be fine.* (iFraP1)  *cause of the circumstances I think that it’s the safest way to do it and the only way to do it, and we haven’t got really much choice* (iFraP3)  *I was quite shocked really. It didn’t help that the GP only told me over the phone (…) he said ‘oh by the way you’ve got osteoporosis. The good news – it’s in your spine, but the good news is it’s not in your hip. And I’m going to prescribe you these tablets anyway’. So I was a bit shocked and I felt it was a bit blasé the way you know, the way I was told*. (B073p)  *[Why patient requested a face-to-face appointment after an initial telephone conversation] Well, when you read the list that comes with the tablets, it’s quite horrifying the symptoms you can have. So I really wanted to know about what it could do to my body. So I think that was the reason.* (B022p)  *I don't always feel the need to go in and see anyone face to face. It’s just getting the information you want answered, answered immediately.* (B013p)  *In actual fact, when they first phoned me about it, I couldn’t understand why because it was actually my cancer consultant at the hospital who sent me for the scan and I couldn’t understand why my GP was ringing me and, to start off with, I was really, really worried because I thought “oh please don't tell me the cancer has gone to my bones”* (B006p) |
| **Perceived effectiveness**  *The extent to which the remote consultation is perceived as likely or unlikely to achieve its purpose* | *I just think sometimes that’s all that’s needed [a remote video consultation], you don’t always need the doctor, the consultant to lay their hands on, you just tell them what’s happening and then they might have to send you for a test but it’s not always immediate so if you’re on a video call you could get to the exact same position* (ERAP13-2)  *I mean for me it was more convenient because I could have the conversation I needed to have quickly and efficiently, but no that hasn’t been a problem for me personally at all* (ERAP1-1)  *I think in lots of ways it’s better because it’s quicker, you don’t have to leave your home, things run on time and it’s efficient* (ERAP1-2)  *they used to provide me with blood cards and I’d go and get my blood tests done myself and then I’d either email or ring in and someone would call or email me back with my results, so it didn’t actually need in-person appointments* (ERAP3-3)  *So some things you can talk about over the phone and sometimes it’s the reassurance you know it’s having somebody there that you can just say I’m struggling. You might just say something like ‘don’t take that tablet try taking one of these you know a Naproxen or something which I can’t take but’ they just might tweak something slightly that just fits the bill and it’s good, I think it’s good to have them there that they can talk to you. You don’t always need a face to face no I agree with that* (ERAP6-3)  *I have always been hostile to the telephone call (…) face to face is, and I genuinely mean this, infinitely better than a telephone call.* (ERAP9-2)  *if it could be dealt with over the phone and a lot of things can or video as people have got a lot more comfortable with Teams and Zoom and things, I’d never even heard of those things before, it’s not as good […]* *you can’t replace the face to face where they have to look at your joints and feel your joints and see what movements are hurting and that sort of thing.* (ERAP1-3)  *you can’t beat the face to face* (ERAP1-2)  *I think if it was the first appointment or an early appointment that would be very difficult on both parts you know for the consultant as well really* (ERAP1-2)  *I have actually seen my Rheumatologist as well face to face, it wasn’t even a question she didn’t say which would you prefer she just said oh I think I need to see you and I went, there was nobody else in the waiting room, I just went straight in, at a clinic they do a local outreach clinic so that was good, cos she needed to see my joints really and examine me properly* (ERAP1-3)  *I think a video consultation would be better than just a telephone consultation because If I’ve got any issues or anything I could show the doctor I could show it on a video but trying to explain on a telephone wouldn’t be as easy or as useful* (ERAP13-1)*.*  *I don’t, I don’t I can’t see how they can possible guide you without seeing you or, so it’s not been very successful for me to be honest* (ERAP6-1)  *Face to face is, and I genuinely mean this, infinitely better than a telephone call. They are utterly incomparable, the telephone call is an emergency option it is not a valid way of conducting a health process because Rheumatoid Arthritis particularly and so many other conditions demand a holistic approach they do not demand a telephone approach.* (ERAP9-2)  *So I believe if you do go to something like the remote consultation they should be significantly more frequent because that gets around this idea of if there is a deterioration you can feel cut off* (ERAP9-1)  *I think there is a danger that you maybe under play things on the phone, it’s quite difficult to really tell people how you are feeling* (ERAP1-1)  *I don’t know what that person at the other end of the phone is doing are they frowning, is it a bit of a sneer, I’m not suggesting it would be but it could be whereas if I’m looking at them I can see their eyes, I know what’s being said. Also you can’t establish, sorry I don’t feel that one can establish a good relationship over the phone. Relationships come from interpersonal interaction and that happens in a similar space, it doesn’t happen at a distance.* (ERAP9-3)  *I would be quite happy to have a telephone conversation with Dr rather than go to the clinic because very often I go to the clinic I don’t need him, if I don’t need him for anything if I’m fine and things are carrying on perfectly normal and nobody’s changing anything then I’d be quite happy to have a telephone conversation with him* (…) *I know not everybody’s like me I’m just speaking for myself now, if I really did need to see Dr X even if I’d got a telephone consultation with him but had a really bad flare up or something happened that I really wanted to see him then I would contact the hospital and say I’d got a telephone consultation but I really need to see Dr X face to face this time, I would do that and hopefully they’d comply, but other than that I’d be quite happy to talk to him on the phone* (ERAP11-3)  *Well you’re sitting with someone that’s got all your, well I’m sure they are on the phone, but they’ve got your whole history you’re big fat file is there and they know and they’re flicking backwards and forwards and checking dates and when something flared up and something didn’t and what happened and the treatment before (…) on a telephone call you know she might not have said (referring to the rheumatologist) I think it’s time for Anti TNF, she may have done but I can’t see it being so constructive as seeing someone face to face* (ERAP10-3)  *Well, yeah, you see who you’re talking to [yeah]. Yeah, I think that would be better, yeah. (…) ‘cause it’s like you’re actually seeing someone, aren’t you? Yeah, and that would be all right, yeah.* (iFraP3)  *I still think face to face* (iFraP1)  *I think face-to-face, this is going back, I think the face-to-face is much better than over the phone* (iFraP3)  *Over the telephone would be more difficult because I think some of your things are visual and I think that was an advantage but I think over sort of on-line, Zoom or whatever, would certainly be of benefit when you could see those.* (iFraP1)  *If you go to your GP, you expect them to tell you those sorts of things and maybe that's where I've been a bit foolish in having a telephone conversation as opposed to going, and seeing, and telling them my viewpoint in these things.* (iFraPFG)  *I think if I’d been approached by telephone I would have been, I think I’d have been less keen to have engaged. (…) If I’d wanted an excuse it would have been easier to have given them an excuse not to do it (…) I think it’s just the remoteness from the hospital environment for me.* (iFraP4)  *No, nothing. “You're just taking some tablets”. Didn’t tell me what I was taking. I just went along and took it, you know, went to collect the prescription, but I did ask the pharmacist…* (B061p)  *you just can’t – you don’t think of the right range of questions when you’re on the phone and getting that information. If I’d been sat in the GP’s surgery with him, I think I would have been a bit calmer and more focused, than talking to me on the phone like that. So I would definitely like that to be the situation* (B073p)  *(Reflecting on the GP) And so obviously then we had a good talk actually on the phone even then and yes she gave me the formal diagnosis* (B034p).  The same participant then reflects on a telephone consultation with a private consultant:  *I had to have a consultant referral, but he was a rheumatologist because that’s what they wanted and frankly he was useless, completely useless and he was on the phone you know because then we were in lockdown. So that was kind of disappointing.* (B034p)  *I probably wasn’t hearing her properly either because we were outdoors you know and the wind interferes with a phone conversation. It was the wrong place to be having a conversation like that {diagnosis}* (B017p)  *I think telephone access is brilliant to both – to anybody medical. I don't always feel the need to go in and see anyone face to face* (B013p)  *Especially at the moment it’s only telephone calls which up to an extent, they can be very good. But I don’t think there’s anything that can replace a face-to-face consultation because you can tell an awful lot from a person’s face* (B033p) |
| **Lack of self-efficacy**  *The patient’s lack of confidence that they can perform the behaviour(s) required to participate in a remote consultation* | *I’m of sound mind as I can explain, but I’m sure there’s loads of people out there that can’t explain exactly what their situation is and it has to be a visual thing* (ERAP6-3)  *Well it gives you confidence that you can actually see the person and they can see you and how bad you are, it’s hard to explain to somebody how things are aching and it hurts when you move* (ERAP8-1)  *I think a video consultation would be better than just a telephone consultation because If I’ve got any issues or anything I could show the doctor I could show it on a video but trying to explain on a telephone wouldn’t be as easy or as useful* (ERAP13-1)*.*  *I think there is a danger that you maybe under playthings on the phone, it’s quite difficult to really tell people how you are feeling* (ERAP1-1)  *It’s just I get a bit panicky…when my phone goes off. No, not a text. I’m fine with texts and photos and videos, WhatsApp, I’m fine with that. It’s just what they do, like… No, they, that’s what that would be, wouldn’t it? Just sending it to your phone. Yeah, could do that, yeah. Yeah. I’m getting there [laughter]. I’m getting there with phones. If it actually rings I go into a panic.* (iFraP3)  *There are times when she [mother] will probably struggle with the telephone because of hearing, but she certainly wouldn’t be comfortable with video I don’t think* *sometimes telephone is difficult and, well, she just doesn’t do technology so, you know, she has no experience of video links so she would feel very tense in that situation, I think.* (iFraP4)  *People would struggle with the technology of course, that might be difficult [yeah] not everybody’s computer literate (…) you’re talking about an older population here aren’t you? so they could have hearing problems, video problems, sight, you know, can’t see* (iFraP2)  *…you just can’t – you don’t think of the right range of questions when you’re on the phone and getting that information. If I’d been sat in the GP’s surgery with him, I think I would have been a bit calmer and more focused, than talking to me on the phone like that. So I would definitely like that to be the situation* (B073p) |
| **Self-efficacy**  *The patient’s confidence that they can perform the behaviour(s) required to participate in a remote consultation* | *I’m not one of the most confident IT users I’m in the wrong age group really to have grown up with it, but I’m fairly comfortable with it because of the work that I’m doing through MS Teams and Zoom because of the work that I’m doing* (ERAP2-3)  *if it could be dealt with over the phone and a lot of things can or video as people have got a lot more comfortable with Teams and Zoom and things* (ERAP1-1)  *I’ve become used to doing things like that [being texted information by clinician], receiving things by different methods, so it’s, yeah, it’s reasonable easy to pick up new ways of doing things like that.* (iFraP4)  *After the last few weeks of Zoom and WhatsApp video I don’t think I’d mind. Prior to that I might have said that I preferred the telephone [laughs], but I don’t think I’d mind now* (iFraP4) |
| **Opportunity costs**  *The extent to which benefits, profits or values must be given up to engage in a remote consultation* | *had I been really struggling I think I would have had to say ‘look I need to see somebody or maybe a face time or somebody needs to see my hands’, does that make sense?* (ERAP14-1)  *They do like a DAS score where they will manipulate your joints and you can kind of say about the level of pain, well obviously that’s a bit of a no no over a telephone consultation* (ERAP4-1)*.*  *I mean obviously for some things it wouldn’t be would it because you’d need to be examined, you know you’d need them to see you* (ERAP1-2)  *they can’t then feel your joints* (ERAP3-1)  *have a lesion on my back and unfortunately, I don’t possess a selfie stick or anything and couldn’t work out how to put my phone in the right position so the doctor could see a video of my back, so that ended up in a face to face consultation, or back consultation.* (ERAP5-3)  *Well it gives you confidence that you can actually see the person and they can see you and how bad you are, it’s hard to explain to somebody how things are aching and it hurts when you move* (ERAP8-1)  *you can’t replace the face to face where they have to look at your joints and feel your joints and see what movements are hurting and that sort of thing.* (ERAP1-3)  *Tomorrow I’m going to actually see the consultant because obviously they can’t feel your joints can they over the phone* (ERAP1-2)  *You need to be seen by a consultant cos if they’re actually like touching your feet or looking at your hands they actually need you in person* (ERAP3-3)  *One of the main things is that they do check your joints and you can’t do that over a screen* (ERAP3-2)  *I don’t, I don’t I can’t see how they can possibly guide you without seeing you or, so it’s not been very successful for me to be honest* (ERAP6-1)  *But it is nice to see, obviously you have to see a consultant in person cos they have to actually properly look at you and you can’t do that on the phone* (ERAP3-3)  *I think maybe it’s part of the human psyche but if you’re seeing a person face to face you do feel like you’re being more looked after even if that’s not necessarily true it’s your perception maybe rather than the reality but sometimes perceptions more important than reality isn’t it. You do feel like you’re more of an individual and getting more of a service just due to the fact that you’re there* (ERAP4-3)  *I’m quite happy to talk to my GP but I’d much prefer to see my GP because things crop up during a conversation I find when it’s face to face, whether it’s a GP, the dentist or even family things crop up during conversation because of a stimulus that that face to face has created, whereas I find generally speaking telephone calls or virtual platforms just reduce that personal touch.* (ERAP2-3)  *if I was speaking with Dr [name] tomorrow on the phone she would say, and how are you and I would say I’m fine because that’s my go to which is ridiculous I know but that’s what I would say, and quite often I do that just without thinking when we go for an appointment and I’ll go yes I’m fine and [partner] will be there and say well actually you’re not, but also she can see me and she can see I’m not so I think there is a danger that you maybe under play things on the phone, it’s quite difficult to really tell people how you are feeling* (ERAP1-2)  *but you can’t show the pain on somebody’s face because most people if you say how you doing will say I’m fine I’m ok but you’re looking at them and you can say well you don’t look ok* (ERAP8-1)  *about 3 or 4 years ago I actually went up to see the doctor and I felt terrible that day everything was hurting, so I was in the waiting room and your doctor says ‘[name] can you come in’, so I struggled to get in and sat down and he says ‘you’re not doing well today are you’, and he’d seen that I was in a bad way without me even saying anything so I think sometimes seeing somebody gives you more information that trying over the phone* (ERAP8-1)  *Rheumatoid Arthritis particularly and so many other conditions demand a holistic approach they do not demand a telephone approach. Now the telephone approach might be part of that holistic approach but if one denies the face-to-face, one denies the patient of that holistic view on the behalf of the consultant or the nurse* (ERAP9-2)  *I don’t know what that person at the other end of the phone is doing are they frowning, is it a bit of a sneer, I’m not suggesting it would be but it could be whereas if I’m looking at them I can see their eyes, I know what’s being said. Also, you can’t establish, sorry I don’t feel that one can establish a good relationship over the phone. Relationships come from interpersonal interaction and that happens in a similar space, it doesn’t happen at a distance.* (ERAP09-3)  *I think GP’s learn a lot from body language it isn’t just about what’s being said the verbal queues necessarily a lot can be taken from body language and I think that’s an aspect that obviously with the best will in the world remote consultations will never supersede that, so I don’t think remote consultations will ever replicate that and the relationship that people have with their Practitioners as well I think you’d lose that immensely if you just do remote consultations (…) I think not only from the patient’s point of view but also the General Practitioners too, I think they will lose or it will be part of their skill set that’s almost weakened if they’re not seeing patient’s actually seeing them face to face on a regular basis* (ERAP4-3)  *you can’t replace the face to face where they have to look at your joints and feel your joints and see what movements are hurting and that sort of thing.* (ERAP1-3)  *the drawback I would think would be if they wanted to inspect your joints for inflammation which the doctors do they have a look, so they couldn’t really do that* (ERAP7-3)  *It is this need to, this psychological need to be looked after and part of being looked after isn’t just hearing someone’s voice it’s seeing their eyes, it’s seeing their lips, it’s looking at the smile, it’s the interaction that’s between the clinician and the patient* (ERAP9-3)  *Yes especially with arthritis because they’ve only got to look at you and they’ve only got to touch your hand or your fingers and if it’s hot they know it’s on the go its active* (ERAP8-3)  *Now on the phone you know I like to think I sound quite a chirpy person, but you can’t tell if I’m really struggling to sit down or stand up you know* (ERAP14-3)  *A few years ago I went to see Dr X at the Hospital and I was on a real bad day and everything was aching and everything was bad, and he came over to shout us and I got up and walked in and before I sat down he said you’re not well today are you, so he’d seen that from me just walking in that I was suffering whereas over the telephone you can’t see that, and people say oh how are you and you say I’m fine but you’re not but you automatically say I’m fine. So I think the phone sometimes a lot of people will, especially the older people, will slip through the cracks cos people normally just say I’m fine and everything’s not (…) its much easier to say over the phone that your fine as opposed to looking somebody in the face and them looking back at you thinking yes I’m saying I’m fine but really I’m not. They can read you I think can’t they* (ERAP8-3)  *one of the lady doctors actually rang me at home to see how I was, which very good, good appointment but normally you would have your joints tested and they would see that everything was ok, but I suppose you do miss that and you think well I haven’t had that done now for well it’s two years over two years now, but on saying that I’m alright but I suppose if I was really poorly you’d be a bit concerned wouldn’t you only speaking on the phone.* (ERAP10-3)  *I think it’s [face to face] slightly easier to read body language, it’s slightly less staccato, there’s slightly less of a jump, it just, I don’t know, it feels more focussed somehow.* (iFraP1)  *Face to face can be quite important can’t it because you know the doctor, you walk into the surgery and the doctor immediately can read from the way in which you’re conducting yourself* (iFraP2)  *Well, if you could see them on the video but otherwise you, like now, just on the normal telephone you need it in front of you, don’t you, printed out because I think just telling the numbers, you know, like you say, oh, well, it would avoid 10 more breaks, it doesn’t mean to you as the visual aid does it. The visual aid is much better in front of you to see than someone telling you something (…) if you’ve got a screen with it on but just to be told it doesn’t really sink in, does it? Not like a visual aid..* (iFraP3)  *depending on how the consultation went, you might have to share with the person some written information of some kind which might be difficult (*iFraP2)  Although RC wasn’t always seen as offering an appropriate forum for discussing issues and concerns, there was an acceptance that this was the way it had to be at the moment i.e. acceptable in the pandemic – (*Interviewer) Yeah, and do you find a telephone consultation is adequate for discussing concerns and issues? - No, but that’s the only option open.* (B070p)  *But I don’t think there’s anything that can replace a face-to-face consultation, because you can tell an awful lot from a person’s face* (B033p) |
| **Decreased burden**  *The perceived decreased effort that is required to participate in a remote consultation* | *I don’t have to sit and wait for about 2 hours like I normally did so that was good, and I don’t have to travel* (ERAP1-1)  *I mean for me it was more convenient because I could have the conversation I needed to have quickly and efficiently, but no that hasn’t been a problem for me personally at all* (ERAP1-1)  *you haven’t got well the time off work, you haven’t got the travelling up there, the waiting around with people and all that you know it’s a matter of a telephone call that you can do in your break you know I just come off the computer and speak to her cos I’m here at hand* (ERAP6-1)  *at the moment if they [clinician] see somebody they’ve got to do a big clean before the next person can come in but when you’re on the phone you haven’t got that issue* (ERAP13-3)  *hasn’t now got to get on a bus and spend you know an hour travelling and then all the bus fares that go with it the waiting time has gone hasn’t it or potentially gone (…) you know the number of people that I used to hear complaining when I was sitting in the waiting room you know ‘I’ve been here 25 minutes now past my time you know blah blah blah’”* (ERAP2-1)  *we [GP] actually have had more phone and email contact than actual in person, cos sometimes it’s quicker and it worked for me* (ERAP3-1)  *I think in lots of ways it’s better because it’s quicker, you don’t have to leave your home, things run on time and it’s efficient* (ERAP1-2)  *Rather than have to drive across the city and get stuck in traffic, having a phone call with someone could be a lot quicker especially if you were just nipping up there for something quick* (ERAP3-1)  *I think if you go somewhere and you have to wait as often the waiting times are really quite long, I think that when I used to go up to [hospital] often you’d wait for a good hour before your appointment time, it’s not their fault but they often do get behind, and then you’ve got to travel there and then wait and then have it and then travel back so it takes a morning out really whereas this way [telephone] it’s a lot quicker and its better for people that work. I think if you don’t work it doesn’t matter but if you’re trying to fit it in around work I was often having to book a half a day holiday or make my time up because I was just waiting around for appointments* (ERAP3-3)  *The situation of having to wear a mask and wear gloves and sanitise and all this that and the other, that’s gone so I think it puts people able to take a sort of deep breath and a sigh to think ‘well I haven’t got to go through all this rigmarole’* (ERAP2-1)  *I really don’t like hospitals, I think they are just bug factories so telehealth for me is an absolute boom* (ERAP5-1)  *there’s obviously a lot less risk cos your just at home* (ERAP1-2)  *I think for people who are able to evaluate to some extent whether they need to be seen or not (…) then I think it’s a good thing it saves a lot of time and hassle, it saves you going down to the surgery that’s full of people and you can be fairly quickly expedited* (ERAP12-3)  *people are getting busier and busier these days too, so rather than going and losing an hours work and maybe have to ask the boss for time off etc. and go to the surgery, if that’s able to be done effectively via that kind of medium then yes there’s certainly value in that.* (ERAP4-3)  *Obviously, I can see the benefits and the time benefits and everything else and also that thinking it through a bit further the benefits of doing it in your own home without having the, you know, the travelling and trying to find a parking space before you get there and all the rest of it.* (iFraP1)  *It makes it an awful lot easier for people, doesn’t it, that they can just be sitting waiting at home rather than sitting and waiting in a waiting room, and you haven’t got to worry about the transport and getting there and back for those, for whom that might be a worry or concern. So yes, in many ways it is easier.* (iFraP4)  *Some people don’t have mobility, it’s a bit of a trial, you know it’s quite a long way to go to the fracture clinic isn’t it, it’s a good car ride from where I am and with public transport not being particularly good, a bit problematical, and also it must be more efficient* (iFraP4) |
| **Increased burden**  *The perceived increased effort that is required to participate in a remote consultation* | *it’s hard to explain to somebody how things are aching and it hurts when you move* (ERAP8-1)  *I think a video consultation would be better than just a telephone consultation because If I’ve got any issues or anything I could show the doctor I could show it on a video but trying to explain on a telephone wouldn’t be as easy or as useful* (ERAP13-1)*.*  *I think there is a danger that you maybe under playthings on the phone, it’s quite difficult to really tell people how you are feeling* (ERAP1-1)  *There are times when she [mother] will probably struggle with the telephone because of hearing, but she certainly wouldn’t be comfortable with video I don’t think* *sometimes telephone is difficult and, well, she just doesn’t do technology so, you know, she has no experience of video links so she would feel very tense in that situation, I think.* (iFraP4)  *People would struggle with the technology of course, that might be difficult [yeah] not everybody’s computer literate (…) you’re talking about an older population here aren’t you? so they could have hearing problems, video problems, sight, you know, can’t see* (iFraP2)  *I think potentially there’d be problems… large documents on a small smart phone screen, I’d need to have two laptops side by side [they both laugh] which I don’t think I could do, not many people would I don’t think, no I think there would be problems with that, yeah, unless the person at the other end was pretty clever on these things and knew how to share screens or something, I don’t know [yeah] that’s beyond my technical abilities.* (iFraP2)  *It’s difficult because I know that my body and the way I react is an issue which is, you know, doctors can only offer what they can offer…So I understand that but I really have no idea what the next medication will be and when that’s going to happen. All I ask is that at all possible, I can see the doctor face to face because I believe that it’s easier to deal with it face to face…But you know, that may not be until November and it may not happen.* (B067p).  *…when somebody’s given a diagnosis or news that they struggle to cope with, you don’t hear anything beyond processing “Blummin’ heck, I’ve got a diagnosis of osteoporosis”. I was so shocked, I don’t think I heard anything else in the phone call. She should have told me that “We need to discuss the results” which would have given me some indication. I’d have thought “Hmm, she needs to see me. This isn’t looking good” and then I would have been prepared and had a proper discussion with her* (B017p)  *I wasn’t hugely thrilled that when I got the – he rang me on my mobile and I was on a day out with a friend in [Place 1] and walking round [Place 1] suddenly get this information that perhaps was a bit suboptimal!* (B012p)  *I was quite shocked really. It didn’t help that the GP only told me over the phone, and I was out and about and he rang me and he said ‘oh by the way you’ve got osteoporosis. The good news – it’s in your spine, but the good news is it’s not in your hip. And I’m going to prescribe you these tablets anyway’. So I was a bit shocked and I felt it was a bit blasé the way you know, the way I was told.* (B073p) |
| **Ethicality**  *The extent to which remote consultations had good or poor fit with an individual’s value system* | *you don’t always need the doctor, the consultant to lay their hands on, you just tell them what’s happening and then they might have to send you for a test but it’s not always immediate so if you’re on a video call you could get to the exact same position then go for this appointment where you have to have and x-ray or scan.* (ERAP13-2)    *I think the telephone or video consultations would be a good thing and allow the doctors to see more people* (ERAP13-1)  *Having a video call wouldn’t actually make it worse, they would be able to see more people* (ERAP13-2)  *Yes I still think it’s a really a good idea especially if it means that the doctors or consultants can see more people you know throughout the day. Obviously at the moment if they see somebody they’ve got to do a big clean before the next person can come in but when you’re on the phone you haven’t got that issue* (ERAP13-3)  *if it could be dealt with over the phone and a lot of things can or video […] it’s not as good but if it’s going to be quicker rather than waiting 6 months to see somebody which a lot of people have to, if you can have a quick video consultation it’s much better for everybody* (ERAP1-3)  *if you can do video calls or phone calls then leave the other calls for people that need to be there. Like people that have got a new problem that needs looking into* (ERAP13-2)  *some people aren’t comfortable on the phone, you know maybe it’s not ideal for everybody* (ERAP1-1)  *I’m sure there’s loads of people out there that can’t explain exactly what their situation is and it has to be a visual thing* (ERAP6-3)  *I am very aware that the silver surfers i.e. my age group and maybe the 65 plus people are a little bit reticent and a little bit frightened of getting involved for the first time* (ERAP2-3)  *if it could be dealt with over the phone and a lot of things can or video as people have got a lot more comfortable with Teams and Zoom and things, I’d never even heard of those things before, it’s not as good but if it’s going to be quicker rather than waiting 6 months to see somebody which a lot of people have to, if you can have a quick video consultation it’s much better for everybody* (ERAP1-3)  *I think that would be a useful tool actually because there are so many people who’ve got it now and very often you’re taking up and appointment and you don’t really need to go there and take up that time* (ERAP11-3)  *I’d be quite happy. My mother wouldn’t cope (…) I’m afraid this is one of my hobby horses at the moment, that we’re all being expected to be computer literate and many people over 80 aren’t and are feeling slightly excluded* (iFraP4)  *I think you’d always have to maintain the option of a face to face somehow for people who can’t cope with technology.* (iFraP2)  *I rang the GP back and again they gave me another me another telephone call. At no point did I see the GP and all they reiterated was ‘yes, it’s osteoporosis, Alendronic Acid is what you need and there is a prescription ready and waiting for you’…So I didn’t take it up at that point. I started to get quite upset about it because I felt that I’d been diagnosed without a lot of support.* (B032p)  *but feeling that to be given a diagnosis over the phone was atrocious. And I know now that lots of people have that experience. And then to take, with a phone conversation, to take a drug that has such systemic sort of impact.* (B017p)  *I’ve phoned, I’ve written yesterday a note to the GP, who was going to ring me this morning because I want to be referred again. I’ve got lots of questions, and I don’t see why, having had a diagnosis, and not being told all of these things, why I shouldn’t have a face-to-face referral to the consultant.* (B040p) |
| **Positive affective attitude**  *Positive feelings about remote consulting* | *if I phone up and they say we’ll phone you back or you know we’ll do a video call this afternoon ‘bloody hell that’s great’* (ERAP5-1)  *it was fine for me* (ERAP1-1)  *I was very happy and satisfied that everything was A Okay.* (ERAP4-1)  *I’m quite happy with it really* (ERAP3-1)  *A lot of people I know whinge about having to have phone calls but I don’t, I find them fine.* (ERAP10-1)  *I’m quite happy with phone calls or teleconferencing.* (ERAP5-2)  *I had a telephone call from a consultant which I found very useful, quite happy* (ERAP10-1)  *No I was quite happy with doing the telephone thing with the consultant, he actually rang me back anyway and filled me in with what he thought at the time* (ERAP8-2)  *If it was something that could be discussed over the telephone well that’s fine with me* (ERAP15-2)  *I think the remote consultations, well I personally believe they’re a great idea and technology or the technologists have stepped up to the mark in huge numbers and cracked a lot of problems* (ERAP2-1)  *I feel safer at home, so I would prefer to have a phone call from the consultant and have a chat* (ERAP15-1)  *So I think, for me, they’re going to be a terrific tool going forward* (ERAP2-1)  *the introduction was there and he was asking all the right questions and it was you know I felt as though I was being looked after* (ERAP9-1)  *recently going into a waiting room where there are maybe 5 people in a space for 20, bloody glorious, so consultations any sort of phone, video consultation absolutely great* (ERAP5-3)  *For me personally I don’t think there was anything really missing from it. (…) she covered everything over the phone, cos knowing it was coming up you know what questions she’s going to ask you, you know your height, your weight and everything else so I got everything all ready so that I’d got the answers for her so it went very very easy, so I’d not got any problems with that whatsoever* (ERAP15-3)  *I’ve just had the [telephone] follow-up from the [hospital], which was, I thought was quite good.* (iFraP3)  *Yes, I would be happy for a telephone consultation [with GP]* (iFraP4)  *I’m quite happy doing this on the phone. I’d probably be equally happy doing it by video, but no, personally I can’t see any other reasons for not doing it.* (iFraP4)  *Yeah I mean I think video, you know, Zoom or whatever, it’s really good, everybody’s just discovered Zoom, I mean we have Zoom meetings now with groups that I’m in and we’re very impressed with what we can do with it (*iFraP2)  *we now do have access to video links, increasingly, which are pretty good, then I would have thought it has great advantages (…) so yeah I mean I think it’s the way forward.* (iFraP2)  *I think telephone access is brilliant to both – to anybody medical. I don't always feel the need to go in and see anyone face to face. It’s just getting the information you want answered, answered immediately.* (B013p)  *Oh yes, because it was just like having a consultation in the clinic. It was fine.* (B057p) |
| **Negative affective attitude**  *Negative feelings about remote consulting* | *you can feel cut off* (ERAP9-1)  *all the local GP’s were basically shutting the doors and that was it you’re going to have to fend for yourselves (…) At first I thought I was just going to be left on my own to fend for myself, if it flared up tough, but like I say the doctors closed their doors didn’t they when it first started but the [hospital] didn’t so that made me feel better* (ERAP8-3)  *I did not like it (…) as soon as the opportunities for face-to-face contact re-arrive I believe we should firmly go back to that. I have always been hostile to the telephone call (…) face to face is, and I genuinely mean this, infinitely better than a telephone call.* (ERAP9-2)  *you can’t beat the face to face* (ERAP1-2)  *I think the future is going to be a mix of remote and face to face contact with General Practitioners and indeed others, but I can’t foresee, well I don’t think it would be healthy just to do remote consultations.* (ERAP4-3)  *I still think face to face* (iFraP1)  *I think face-to-face, this is going back, I think the face-to-face is much better than over the phone* (iFraP3)  *to an extent you know face to face is better than not face to face and I know the video links are good but they’re not perfect* (iFraP2)  *but feeling that to be given a diagnosis over the phone was atrocious.* (B017p)  *It was the wrong place to be having a conversation like that* (B017p)  *you just can’t – you don’t think of the right range of questions when you’re on the phone and getting that information. If I’d been sat in the GP’s surgery with him, I think I would have been a bit calmer and more focused, than talking to me on the phone like that. So I would definitely like that to be the situation* (B073p) |
| **Key:** ERAP1-1 (ERA study, participant 1 - interview 1/2/3); iFraP1 (iFraP study, participant 1); B013p (Blast Off participant 13) | |
